# A Sequel to an Essay on the Yellow Fever, Principally Intended to Prove, by Incontestible Facts, and Important Documents, That the Fever, Called Bulam, or Pestilential, Has No Existence as a Distinct, or a Contagious Disease

**Published:** 1818-02

**Authors:** 


### PART II.

### COMPREHENSIVE ANALYTICAL REVIEW OF MEDICAL LITERATURE.

"Tros, tyriusve, nobis nullo discrimine agetur."

*A Sequel to an Essay on the Yellow Fever, principally intended to prove, by incontestible Facts, and important Documents, that the Fever, called Bulam, or Pestilential, has no Existence as a distinct, or a contagious Disease.* By Edward Nathaniel Bancroft, M. D. Fellow of the Royal College of Physicians, Physician to the Army, and late Physician to St. George's Hospital. London, 1817, 8vo. pp. 487.

The Medical History of our West India possessions presents a melancholy detail of a vast destruction of human life from the ravages of the disease which forms the subject of the volume before us; and the painful feelings which the retrospect is calculated to produce, are certainly not lessened by the reflection, that the state of active and protracted warfare in which we have been involved, has, in addition to the other miseries which have flowed from that source, principally contributed to swell the catalogue of victims to this scourge;—that many thousands of our brave countrymen have escaped the fury of battle, and all the varied dangers "per mare, per saxa, per ignes," incidental to the life of the soldier and sailor, only to fall an inglorious sacrifice to this insatiate foe! Nor have its visitations been limited to the transatlantic shores alone; the inhabitants of many of the southern parts of Europe have, on various occasions, felt severely the pressure of affliction and mortality from this widely extended cause. While in common with every feeling mind, we regret the discrepancy of opinion respecting its origin and nature, which has prevailed among the only legitimate judges of the question, and condemn the asperity and intemperateness in which the contending parties have too frequently indulged, we cannot but rejoice in the prospect which now opens on us, of the discussion being at length brought to a speedy termination. The overwhelming mass of evidence which Dr. Bancroft has now brought forwards, in disproof of the existence of contagion in Yellow Fever, will, we confidently anticipate, put to flight a chimera, which has in too many instances seduced the attention from the true sources of the disease. The periodical publications, it is true, have lately teemed with refutations of the doctrine of Contagion; but in the fleeting and insulated form of those communications, much of their weight and authority is necessarily lost. We, therefore, hail with real satisfaction the appearance of a work containing an invaluable store of original and highly respectable documents, collected and arranged with no ordinary research and ability, and supported by argumentative talents of the first order: And we trust we do not deceive ourselves in hoping, that the discordant clamour with which the public ear has been so long assailed, will be now reduced to silence; and that we shall yet enjoy many halcyon days of peace and goodwill, after a contest exhibiting so irreconcileable a character, and conducted with such pertinacity, that an uninterested observer might have justly exclaimed with our great Moralist on a similar occasion, "The Inquirer is kept in continual suspense, and by a kind of intellectual retrogradation, knows less as he hears more."

Since the appearance of the Author's former volume, two publications have issued from the press, in support of the distinct nature and contagious quality of the "Bulam" or Yellow Fever; and by one of the writers a claim has been preferred to the discovery of the alleged peculiarity of its attacking the human frame only *once*. With the view of effecting the subversion of these doctrines, Dr. Bancroft has again entered the Arena; and on all the principal bearings of the question, we conceive that his triumph is complete. The quantity of matter accumulated in the present volume, almost defies an adequate Analysis; but as from the analogy of our opinions on the subject with those of the author, we find very little to oppugn, or to criticize, we shall endeavour to lay before our readers a condensed view of the most important topics under discussion.

We are informed in the Introduction, that the Lords of the Privy Council deemed the opinions of Dr. Pym of sufficient importance to induce them to make application to the College of Physicians for information on the two chief points which he has endeavoured to establish;—the contagious nature of Yellow Fever, and the peculiarity of its attacking only once. The reply of the College, although on the whole favourable to Dr. Pym's pretensions, was undecided, as they properly alleged, for want of experience in the disease. Application was then made by the Council to the Army and Naval Medical Boards. Concerning the communication from the former Board, Dr. Bancroft has not been authorized to give any information. The latter, having collected the opinions of those naval medical officers whose experience enabled them to adduce facts and observations in support, or in refutation of Dr. Pym's propositions, transmitted a concise analysis thereof to the Lords of the Council, together with the original Reports. To these their Lordships has been pleased to allow Dr. Bancroft free access, and from that source a large portion of the evidence contained in this volume is derived.

The author begins his Inquiry by controverting the diagnostics by which Dr. Pym distinguishes his Bulam from the bilious continued, and bilious remittent Fevers; and we are of opinion, that he has undeniably proved that no *specific* difference exists between these forms of fever;—that the points on which Dr. Pym has attempted to found a diagnosis, are merely differences of degree; and, that (excepting the last, the black vomit) they are not peculiar, uniform, nor essential to the fever in question. Indeed, it appears to us, that they obtain more or less in most dangerous fevers, as, we conceive, must be evident not only to all personally and extensively conversant with yellow fever, but even with fever in general; and, further, that Dr. Pym has himself proved the futility, and destroyed the foundation of such diagnosis (if we were to grant his assumption, of which, however, an ipse dixit is the substitute for proof) by asserting, that even Dr. Rush himself mistook the bilious remittent for the Bulam Fever. *(Pym's Obs. p.* 209.

Of these alleged diagnostics, the two first, the appearance of the eyes, and the nature and seat of the head-ache, the author satisfactorily shews, from various authorities, to be vague and indeterminate, and, therefore, perfectly useless in diagnosis. With regard to the absence of remissions, constituting the third diagnostic of the Bulam, Dr. Bancroft adduces a mass of evidence to prove "the simultaneous appearance of both forms of the fever, and their reciprocal *conversions* into each other at particular places and seasons; together with the invariable appearance of remittents at the same places, both *before* the high atmospheric temperature has operated sufficiently to give them the continued form, and also *after* the effects of this high temperature have ceased to exist." Further, Dr. Pym has derived the epidemics of Gibraltar by importation from those of Cadiz, Malaga, and Carthagena, and has thereby identified them with the fevers of those places; and Sir James Fellowes states, that Arejula, Gonzales, and Flores are "the three most eminent physicians in Cadiz, and he believes in Spain." Now, unfortunately for this principal diagnostic, all those writers distinctly mention remissions in their descriptions of the Spanish epidemics; and as regards the fever in Gibraltar, remissions are proved by evidence of seven medical officers of that garrison in the epidemic of 1814. The fourth, or the infrequency and paleness of the yellow colour of the skin, cannot be viewed otherwise than as a relative expression; and it will be sufficient to state, that, from the accounts of Sir James Fellowes, Sir Joseph Gilpin, Mr. Donnet, and others, the suffusion of the skin is observed in every intermediate shade between a lively yellowness, and a dingy, or dark hue. The author also rejects the fifth diagnostic, the duration of the disease, on the principle of the want of uniformity. Dr. Pym says, it runs its course in from one to five days; it is admitted, that it commonly does so in its most aggravated form; but it is proved from Arejula, Sir James Fellowes, Dr. Burnett, Labat, and Dr. Chisholm, that it often continues much longer; further, Dr. Pym states, that "the *remittent* sometimes proves fatal on the second or third day"; and according to Dr. Hunter it even runs its course in twenty-four hours. We have ourselves witnessed death on the third day, in a violent remittent imbibed in the month of September, in one of the most *northern* rivers of the United States. Lastly, respecting the sixth alleged diagnostic, the gangrenous state of the stomach, and the appearance of black vomit, Dr. Bancroft exposes the futility of such criteria, the first of which can only be known after death; and the latter "is the almost unerring harbinger of death." The chief value of a diagnostic is to enable us to ascertain the true nature of a disease; but this refers to its consequences only. Neither is the black vomit peculiar to the continued form; the high authorities of Pringle, Cleghorn, Hunter, Rush and Burnett, prove its occurrence in the remittent.

"I shall only add, concerning this black vomiting, that as it is a mortal symptom, never occurring, it may be said, *in those who recover*, and one which is often wanting among those who die, its appearance in this disease must be much rarer even than death; and this circumstance, joined to that of its *not* being 'peculiar' to the fever in question, render it very unfit to be produced as a diagnostic thereof." p. 30.

Adverting to the inconsistencies contained in Dr. Pym's account of the condition of the pulse and skin, "for which," he says, "the Bulam Fever is remarkable," the Author thus expresses himself:

"Descriptions of symptoms being simply records of natural events in disease, which stand unalterable, however opinions about them may change, will the confusion, the inconsistencies, and errors, every where apparent in Dr. Pym's attempt to frame a diagnosis for the Bulam Fever, be deemed very excusable in one who claims merit for discovering peculiarities therein, which had escaped the sagacity and penetration of all other observers."

We apprehend that sufficient has been said to shew, that the question of the continued form of fever, or the Bulam, is merely one of degree; that the peculiarities which are said to distinguish the Bulam from all other fevers, do not exist; and that, therefore, the supposed distinct fever must be as imaginary as the peculiarities themselves.

The second chapter is devoted to the consideration of other alleged peculiarities, more especially the non-liability to a second attack; which it is stated, was brought under the notice of the Privy Council, in consequence of an application from Dr. Pym.

The merit of originality in this supposed discovery is disputed; Sir James Fellowes awards it to the Spanish practitioners generally; Dr. Pym claims it as exclusively his own, and fixes on the 20th day of October 1804, as the period when that event took place in the garrison of Gibraltar. The security, he represents, to be similar to that which an individual acquires by having undergone the small-pox. Now, Professor Berthe, in his "Precis Historique," &c. published in 1802, gives an extract of a printed letter, dated at Cadiz, May 6th, 1802, in which the writer plainly states, that, like small-pox, after one attack, a future seizure rarely occurs. This opinion, however, the Professor designates as fallacious and dangerous. In the epidemic of Cadiz also in 1800, towards the decline of the fever, the civil authorities of that place grounded their police measures on this opinion:

"Guards were stationed at the gates, to exclude all persons from entering the city, who did not produce certificates of having already had the fever."

Arejula had likewise pointed out the security afforded by an attack of the fever, in the epidemics of Medina, Sidonia, Malaga, and other places in Spain; and states at page 319, that

"At these places, and almost every other, he selected as assistants to the sick, those who had previously undergone the epidemics."

So much for the originality of the alleged discovery, to the credit of which, even had it been confirmed by experience, we apprehend, on the principle of "suum cuique," Dr. Pym had no claim. As to the reality of this supposed "peculiarity," we consider the evidence adduced by Dr. Bancroft from the Reports of the naval medical officers, before adverted to, as well as the result of the examination of the different Journals of naval surgeons, employed in the West Indies, to be perfectly conclusive in the negative. This opinion is corroborated by the answers of five army surgeons, and three assistant surgeons of the garrison of Gibraltar during the epidemic of 1814, to the questions proposed to them by Deputy Inspector Fraser; they all bear distinct testimony to second attacks

We can only briefly notice the author's expositions of the frailty of Dr. Pym's alleged proofs of absolute immunity after one attack. In the instance of the epidemic of Gibraltar in 1804, (on which the supposed discovery seems to have been founded) it is stated, that one hundred and twenty-two men who had escaped the fever, were found on inquiry to have been in the West Indies at some former period, which is inferred to have been the cause of their exemption; and, that the 57th regiment, which had recently served in Trinidad, was introduced into the garrison, during the prevalence of the epidemic, with impunity. These are alleged to be proofs of the Bulam Fever not attacking a second time; but both instances obviously involve the assumption, that all who have visited the West Indies, have necessarily undergone an attack of Yellow Fever;—a fallacy we need not stop to refute. The instance of the men of the 10th regiment, which acquired their security by service in the *East* Indies, is still more palpably defective; because, Dr. Pym having laboured to prove that the Bulam has never appeared in the East Indies, the men of the 10th regiment could not, on his own principles, have obtained their immunity by previous attacks.

Indeed, it appears to us, that Dr. Pym has not steadily contemplated the security, constituting his alleged discovery, in a determinate point of view. In general, he compares it to the absolute immunity which an attack of the small-pox confers; but at other times, he plainly speaks of it as (what in truth it amounts to) merely a relative security; for instance, in his account of the epidemic Yellow Fever of the 70th regiment in Martinique, in 1794, he says, every individual in the regiment was attacked; and, that three officers who had been several years in the West Indies, some time before, had it in so mild a form, as to make it unnecessary for them to be confined to bed:—again, the regiments in Martinique that had been some years in the West Indies, he says, were attacked (in 1794) equally with the corps lately arrived from England; but *with this difference*, that the former "*suffered a comparatively small mortality*:" And further, in the above-mentioned case of the 10th regiment at Gibraltar, he states, that "eight officers who had been in India, were attacked with the fever, and all recovered. Seven officers who had not been in India, had the disease in so different a form, that five of them died." These we take to be fair illustrations of relative security, acquired by habituation to, or seasoning in, a tropical climate; and prove, that in order to obtain such comparative security, it is not necessary that the individual should have passed through an attack of Yellow Fever; while, on the other hand, we may safely trust to the evidence adduced by Dr. Bancroft, to establish that one, or even a repetition of attacks, do not confer *absolute* nonliability.

We have been somewhat diffuse on this point, from a sense of its importance; and because we are anxious to exhibit the merits of the case in as distinct a form as our observation of the subject permits; and we refer to the evidence itself in support of our opinion, that the supposed nonliability to a second attack, so far from resembling the immunity after small-pox, is strictly a relative security, to be acquired as certainly, though more gradually, by tropical residence, (which involves habituation to the remote cause) as by having passed through an attack of the disease;—a condition of the habit which confers security only when the concentration and force of the endemic causes do not exceed the degree to which the individual may have been previously habituated;—and, lastly, a mean of exemption which is liable to be destroyed by (e converso) the regenerated susceptibility which a return to, and residence in, a northern climate effectuate. That the exemption is absolute after one or more attacks, we consider to be perfectly, and most satisfactorily disproved; and we cannot well abstain from expressing our astonishment how Dr. Pym could ever have entertained such an idea, much less have vaunted it as a *discovery;* for very little reflection might have shewn him, that it *could not* have escaped the observation, but *must* have been evident to, and eagerly caught at by those who had passed a series of years amidst Yellow Fever, had such absolute immunity any existence. The facts included in the documents now brought forwards by Dr. Bancroft, will, we cannot doubt, be deemed decisive; and consign to oblivion the premature notion of a discovery in a supposed "peculiarity," which he has proved, does not exist; and which, even for a moment supposing its existence to be any thing more than relative, had been pointed out, and acted on by the Spaniards, many years previous to the 20th of October 1804.

Dr. Fergusson, Inspector of Military Hospitals in the Windward Islands, in his Communication to the Army Medical Board, observes on this point,

"Another piece of doctrine, has been promulgated from the writings of the authors above alluded to (Drs. Pym and Fellowes); that the Yellow Fever cannot be received by the same subject more than once. Of this we again, who live amongst Yellow Fever, not only know nothing, but we see it contradicted by the daily experience of our lives." Page 87.

We have always protested with Dr. Bancroft against the subtilty of making the black vomit a criterion of the Bulam Fever, and regulating the admissibility of the proofs of future attacks by that assumed standard. By acknowledging the legitimacy of such a criterion, as few or none recover after that symptom has appeared, a difficulty, nearly tantamount to impossibility, is incurred, of ever adducing, in the course of even a long life, an unobjectionable instance of a second attack. When black vomit, and its usual immediate sequel, death, take place, the patient is relieved from future attacks of any kind; but in less aggravated forms of Yellow Fever, where there has been no black vomit, and the person has recovered, then, in the event of a second attack, say the advocates for the Nova Pestis, the original one was not a case of Bulam, for one of our diagnostics was wanting; there was no black vomit! — and vice versa. Accordingly, we find this subterfuge incessantly resorted to. Against such sophistry, arguments are vain; and facts, for the reasons we have assigned, difficult to be applied. The Report of Inspector Fergusson from Barbadoes, amongst other cases of second attacks, contains, however, one decisive instance of even black vomit occurring twice in the same individual. A patient of Dr. Caddell, a physician of the greatest experience in Barbadoes, miraculously recovered from Yellow Fever with distinct black vomit, "and died some years afterwards of the same disease, and with the *same symptom*." Against a fact of such decisive import, we know not what reply can be opposed, unless it be, "Non persuadebis, etiamsi persuaseris."

In a rejoinder of considerable extent, Dr. Bancroft adverts to Dr. Pym's examination of the authorities he has adduced in his Essay against the doctrine of contagion. He complains of a disingenuous and partial selection of those athorities for that purpose; and expresses his conviction, that they have passed the ordeal without injury.

"Here Dr. Pym closes the account of what he terms my authorities; and he manifestly intends to have it believed, that he has noticed and refuted *all* those which I had adduced; when in fact, he has completely shunned even the mentioning of nine-tenths of them. The few whom he notices were obviously selected only because they had said or admitted something capable of being distorted contrary to the real and sincere meaning of each; and in effecting this distortion he exults, as 'having by cross-questioning my witnesses, brought out *the truth*,' and 'convicted me upon my own evidence': although in regard to the great body of those who are more properly my witnesses, he is so far from having cross-examined them, that he has not even looked them in the face; and my readers, I firmly believe, will be convinced that he has not been able to invalidate or weaken any *one* testimony or opinion which I had alleged to prove the fever in question to be void of contagion." page 110—111.

A similar complaint is preferred of an equally uncandid and partial selection of some of his evidences against contagion, for the purpose of examination; and the irrefragable character of the remainder is thence very justly inferred. We think it but an act of common justice to Dr. Bancroft, to insert in his own words, the recapitulation of the evidences against contagion, contained in his former volume, which Dr. Pym has not thought proper to oppugn, or even to notice; leaving our readers to draw their own inferences as to the probable motives for such cautious proceeding.

" I have now examined all that in any way merited notice of what Dr. Pym has advanced against my authorities and arguments, with the exception of some circumstances relative to Cadiz and Gibraltar, which are reserved for future consideration; and I cannot but believe that my readers will have been convinced of the fallacy of those principles upon which he has endeavoured to explain, or rather to evade, my inferences, and of the abortiveness of his endeavours to invalidate, in a single instance, either my testimonies or my reasonings. There remains beside a great mass of evidence of which he has studiously avoided even the smallest notice; and this must of course be considered not only as subsisting in full strength, but as having been deemed by him unquestionable and invulnerable: for otherwise, with his dispositions, and the latitude of every kind in which he has indulged, it may be presumed, that it would not have been left without some hostile attempt. To this evidence, therefore, I refer my readers with confidence; and more especially to the very accurate and respectable one of Dr. James Clark, at pages 332, 333, 334, and 760, 761 of my Essay; and that of Mr. Young, Inspector-General of Hospitals, and of all the superior medical officers of the army under Sir Ralph Abercromby in the Windward Islands, p. 334, 335; those of M. M. Desportes and Valentin at St. Domingo, p. 338—341; that of Dr. Hector M'Lean, with the opinions of Drs. Jackson, Scott, Wright, and Gordon, and nearly, if not all, the medical officers of the British army in St. Domingo, p. 341, 342; that of Dr. Hume, p. 346, 347; those of Dr. Walker and of Dr. Grant of Jamaica, p. 350, 351; that of Dr. Ramsay, and of all the medical practitioners of the State of South Carolina, declared unanimously at a General Meeting in Charleston, p. 355, 359; that of Dr. de Rosset of Wilmington, in North Carolina, p. 359; the opinions of Drs. Valentin, Taylor, Hansford, Selden, and Whitehead, in Virginia, p. 360, 362; that of Dr. Davidge at Baltimore, p. 363, 366; that of Dr. Vaughan, in the State of Delaware, p. 367, 369; the opinions of many physicians at Philadelphia, between pages 372 and 386; and at New York, p. 387, 389; and those of Dr. Coit of New London, Dr. Wheaton of Providence, and Drs. Warren and Brown, of Boston, p. 401, 406. I request also the attention of my readers to the facts partly stated, and partly recapitulated between pages 406 and 430; and, finally, to the very important Official Message from the President of the United States on this subject to the two Houses of Congress, p. 430, containing such an uncontradicted and incontrovertible statement of facts, as ought, in every unprejudiced mind, to remove every suspicion of the existence of contagion in the Yellow Fever, at least, in that part of the world." Pages 120—122.

Although Dr. Bancroft considers this quantity of uncontradicted evidence to be "more than sufficient to overthrow Dr. Pym's superstructure, more especially as the foundation of it has been removed in the first chapter of the present publication," he adduces a multiplicity of additional facts and authorities in proof of the local origin of Yellow Fever, and of its being destitute of the quality of contagion. Among other documents, one from New York is not the least curious, which proves from the Contagionists themselves, *that a Fever in every respect resembling the Bulam, prevailed in that city, nearly two years before the arrival of the Hankey at Grenada!* page 124—126.

In illustration of the identity of cause of the continued Yellow Fever, and of the recurrent forms, the following Extract from the Official Report of Dr. Dickson, the late able physician to the Leeward Island Fleet, will be duly appreciated.

"At Barbadoes and Antigua, I had generally seen the disease of an ardent *continued* form, and did not fully understand why authors talked of a Bilious *Remittent* Yellow Fever, until after the capture of the French and Danish Islands. But the anomalies of fever, the shades and changes which it assumes according to the intensity of the exciting causes (which *there* were *purely* and *wholly local)* the state of predisposition, or the spot of residence, could no where be more strongly pourtrayed than in the destructive epidemic of Mariegalante in the autumn of 1808, from the most concentrated marsh miasmata; when the different types of fever were *converted* into each other, of the *worst* and *most aggravated species* I have ever witnessed. Some were affected with the *highly concentrated* Yellow Fever in the continued form; others with *comatose remittents* or *intermittents*, the exacerbations of which were so violent as to carry off a patient in two or three paroxysms; while others, sunk into a low protracted character of fever, resembling typhus." p. 143—144.

After stating the opinions of the Naval Medical Officers who reported on the question of contagion, Dr. Bancroft gives the following summary of them; from which it will be seen that the evidence against contagion is as great and uniform, as perhaps can ever be expected on any disputed point.

"Having stated the opinions delivered in the Reports transmitted to the Privy Council, it may be proper to give a summary of them; and, I will therefore mention that, of the twenty-four Gentlemen from whom these Reports were obtatined, *three* (Mr. Gregory, No. 12, Dr. Kein, No. 15, and Dr. Magrath, No. 17,) have omitted the statement of any opinion on the subject of contagion, as connected with the fever in question; *three* others (Dr. Weir, No. 1, Dr. Blair, No. 2, and Mr. Tobin, No. 21,) have expressed their opinions that *it is contagious; one* of them (Mr. Brien, No. 20,) declares his belief that, in individual or solitary cases, it is 'incapable of communicating itself to those who are contiguous,' but 'that, when several were labouring under the disease at the same time, he believes it to be highly contagious.' And, *another* Gentleman (Dr. Gardner, No. 9,) appears to think, that *local* causes contributed at least as much to the production of the fever in Gibraltar in 1813, as contagion. Of the remaining *sixteen*, the majority have *absolutely* and *positively denied* the existence of any contagious property in this fever; and the rest have declared their belief, that it is not *naturally* or *properly* a contagious disease, although several of them are inclined to believe that it may (as they suppose to happen with most other diseases) acquire a contagious property by crowding, filth, &c. Most of the sixteen gentlemen, who declare that the fever under consideration is *not contagious*, have alleged decisive facts to support their declarations, some of which I have already quoted; and, I shall hereafter have occasion to notice some of the others." p. 178—179.

When we reflect that this evidence in great part proceeds from physicians to fleets, and surgeons of hospitals who have lived among Yellow Fever for a series of years; and, that the Reports here adduced are few indeed, when compared to the great body of medical officers, who, with very few exceptions, we have had occasion to know, are uniformly opposed to contagion; when to these are added the opinions of Drs. Fergusson, Muttlebury, and Adolphus, who have long held official situations of the highest responsibility in the West Indies; when the number and length of service of those who have given their opinions so decidedly against contagion are considered;—the preponderance is so immense, that (as far as the Yellow Fever of the West Indies is concerned) the opposing testimony, when weighed against such a mass of observation and experience, sinks into absolute insignificance.

It would appear, from the Report of the College of Physicians to the Lords of the Privy Council, that they entertain the opinion that Yellow Fever *may* prevail in the British Islands. They express their belief that "the cold of our climate would not prove a preservative against the contagion," (of Yellow Fever) because "it appears that during the months of October and November, when the fever raged at Gibraltar, Malaga, and Leghorn, the temperature was greatly below the average heat of our summer." This inference we beg leave to dissent from; and in extenuation observe, that the College in deducing such conclusion do not appear to have been aware of the necesssity of a certain *preceding duration of high temperature,* which experience proves to be indispensable to the developement of epidemic Yellow Fever. Within the tropics the requisite degree of heat is never absent: and in those places without the tropics which have been occasionally visited by the disease, as North America, and the Spanish Peninsula, the meteorological observations of the various years in which it has prevailed concur in the pre-existence of high atmospheric temperature, for many weeks before the appearance of the epidemics. Temperature to this requisite extent seldom obtains in this climate; and when it does occur, is very transitory. Such evanescent influence is totally inadequate to the production of the disease; and while from insularity, or other causes, our climate retains its *mutable* character, we may, without temerity, discard all apprehensions of the existence of Yellow Fever among us. In corroboration of the steady pre-duration of high atmospheric temperature, as the "sine qua non" of the developement of epidemic Yellow Fever, the following extract from a provincial news-paper is not inapplicable.

"It has been ascertained from tables and records for the last twenty-four years, that in Philadelphia, the Yellow Fever does not prevail when the months of June and July do not exceed 70 degrees; but that in every summer since 1795, when the average heat of these months has exceeded 79 degrees, then the fever has raged; and that it has been most fatal in those years, in which the thermometer has indicated the greatest altitude." *Hampshire Telegraph, November* 1*st,* 1817.

In several of the Reports transmitted to the Privy Council, a belief is expressed that the Yellow Fever, although it does not originate in contagion, or legitimately possess such quality, ***might*** acquire it under accumulation of the sick, and deficient ventilation. The author admits, that the disease may be aggravated by such circumstances; but unconditionally denies the possibility of its acquiring such fortuitous contagious power. On this point, (as far as the ***tropical*** endemic is concerned) we concur with Dr. Bancroft; because, on reference to our experience of many years in the West Indies, we cannot charge our recollection with any instance of Yellow Fever having manifested such contingent property of contagion, *under any circumstances.* One source of fallacious deduction on this point, seems to have been the too narrow limitation of the range of predisposition; for example, a ship enters an unhealthy port; her men imbibe the local noxious exhalations, and are exposed to the other remote causes of fever; she sails with a long list of fevers; the attacks continue at sea, in the order of predisposition, while the local source of the fever has been left behind some hundreds of miles, and is perhaps forgotten; the sick are unavoidably crowded, and at length, in the absence of the original cause, the seizures are ascribed to a contagious property acquired by accumulation; when, in fact, the various periods of attack, should have been referred to the varied degrees of predisposition. In offering this explanation in favour of the ultrà opinion, we merely state the result of our observation. Neither can we admit the justice of the inference, that such alleged contingent property is favourable to the doctrine of a peculiar and distinct disease, the Bulam; which its advocates contend is contagious *ab origine,* independent of those fortuitous circumstances, under which only, some have supposed (not proved) the Yellow Fever to become contagious. Moreover, we imagine, that those most inclined to this opinion, will not agree with Dr. Pym, that it can be conveyed, and re-conveyed across the Atlantic, and from one place to another; because we conceive that such a property, *if ever possessed,* is not of that permanent and imperishable nature to admit of transportation whenever the Contagionists wave their wand; but is dependent upon a casual, local, and transient coincidence of agency; we, therefore, agree with Dr. Bancroft, that it proves nothing in favour of Dr. Pym's view of the subject, its nature or origin.

We are glad to find, that the author has now bestowed due attention on a prolific source of fever under high temperature, the noxious exhalations from the foul hold of a ship. By disregarding this common cause of fever, a contagious origin has been erroneously assigned to fevers, which, making their appearance without exposure to land influence, could not be supposed to have sprung from an endemic source. Of the frequency of such a cause of even the most aggravated Yellow Fevers, no one can doubt after perusing the facts contained in this chapter; to which we are the more desirous of directing the attention of our readers, because we are of opinion, that they will satisfactorily reconcile several seeming instances of contagious fever, with their true origin, an impure atmosphere from the exhalations from a foul hold. It is needless to dwell on the importance of the distinction; the history of the transports from Carthagena, in which the epidemic of Gibraltar in 1810, was reported to have been imported, will hereafter be shewn to be a strong case in point. The accounts of the Regalia transport, and of the Antelope and Childers, ships of war, in which Yellow Fevers of a destructive order recently prevailed from this cause, will be read with the greatest interest. The observations of Dr. Fergusson will shew, that had the Regalia arrived *a year later* in Barbadoes, she would probably have enjoyed equal notoriety with the much calumniated Hankey; the late sickness in that island would have been referred to a second African importation in the Regalia, and error thus confirmed. Dr. Fergusson concludes his observations on this subject with the following important Remarks.

"I am aware how much I have been favoured by circumstances, and what a different interpretation the facts I have collected would have borne, had the present epidemic that now afflicts the islands (1816) broken out in the ordinary course of seasons *a year earlier*, at the time the Regalia was here; my task would then have been a much more difficult one, for these (facts) instead of assisting me to elicit the truth in the manner I have done, would in that case have been turned to the confirmation of error, and the perpetuation of the delusions, in regard to imported contagions." p. 239.

From abundant experience of the danger, we fully coincide with the author in deprecating the practice of heaving down vessels of war, in the West Indies, in the ordinary routine of service at least; as well from the excessive fatigue and exertion it demands, as because it is a process which requires for its execution, local security; or, in other words, a land locked, and therefore, generally, an unhealthy harbour. The instances of sickness and mortality from the effects of clearing a foul hold, in an unhealthy harbour, are numberless; Dr. Bancroft relates a remarkable one, on the authority of Dr. Dickson,

"Of the production of Yellow Fever, accompanied, in twenty-two cases with *black vomit*, and consequent *death*, on board the Circe frigate, principally from the duties of *clearing the hold*, and *heaving down*; by which so many of the ship's company were soon after attacked with this fever, that a hundred and forty-six men were sent to the hospital at Antigua." p. 210.

The fifth chapter refers to the origin of the Spanish epidemics. In speaking of the Peninsula fever, we wish distinctly to state, that our conclusions are drawn from the analogy of the laws of the Yellow Fever of the West Indies, with which our acquaintance has been sufficiently extensive; and as the Contagionists have themselves identified those diseases, we presume the propriety of reasoning by such analogy will not be disputed. By employing the term "marsh miasmata" to designate the exhalations from the soil, to which Dr. Bancroft in his former work, ascribed the Origin of Yellow Fever, he has given his opponents an opportunity of apparently convicting him on his own evidence, by adducing the obvious inference, that where there is no marsh, the Yellow Fever could not have been caused by such miasmata. The topography of some places, where the epidemic has prevailed, as Cadiz and Gibraltar, but where there are no ostensible marshes, has been accordingly exhibited with exultation, as a positive refutation of his doctrine. The error arises wholly from the inadequacy of the term employed to express the origin of such miasmata; and to shew that it is incorrect to ascribe to the author the opinion, that Yellow Fever is always the product of a distinct and ostensible marsh; we subjoin an explanatory quotation.

"In treating of the Ardent or Yellow Fever, as it has occurred at Gibraltar, Cadiz, and other southern parts of Spain, I ascribed its production to the action of those vapours, or exhalations, which result from the decomposition of vegetable, or vegetable and animal matters, in a temperature of not less than 80° Fahrenheit's thermometer, and which are commonly called marsh or paludal miasmata; an appellation which, in compliance with custom, I had occasionally adopted, though I well knew, and had repeatedly declared, that such exhalations or vapours are often emitted from soils and situations which had no resemblance to a *marsh*." p. 253—254.

Again, in a Note, at page 91 of his Essay, he says,

"I beg to state in this place, that, in joining the epithet *marsh*, or *marshy*, to the terms miasmata, exhalations, effluvia, &c.; and in considering these as a cause of fever, *I do not mean to intimate that such miasmata, &c. are emitted solely from marshes (it being certain that they frequently arise from soils in a different state)*; but only to designate *the quality of those vapours*, which are eminently the product of *marshy* grounds."

This ought to have been a sufficient security against the misconstructions which his opinions on this point have suffered. With respect to the existence of paludal effluvia at Cadiz and Gibraltar, he adduces the prevalence during the summer and autumn of remittent fevers at those places, the acknowledged offspring of such exhalations, as indisputably demonstrating their presence and influence, however they may be produced, or from whatever source derived; and as farther proof of the universality of this cause of fever throughout the Peninsula, the statement of Sir James M'Gregor is not irrelevant, which shews, that 22,914 cases of ague were altogether admitted into the British military hospitals in that country.

In the investigation of the alleged proofs of the importation of the various epidemics into Spain, the author has displayed his usual ability and research; and we must observe, that his exposures of the frailties, inconsistencies, and anachronisms, with which those statements abound, refer equally to the proofs of Sir James Fellowes, as of Dr. Pym. Of the first epidemic of Cadiz in 1800, he naturally asks, if the disease is sui generis, and has not appeared for thirty-six years previous to 1800; from whence was it imported on that occasion?

"There must have been somewhere on our globe, a spot on which this disease had existed not long before the time of its supposed importation, and where it was found to possess a contagious power. That they have either proved this, or that there is in fact any such place on earth, I most confidently deny."

We cannot accompany him through his scrutiny of the pretended importations into Cadiz, in 1800, and into Malaga in 1803 and 1804; for these we must refer to the volume itself. The meteorological statements of Sir James Fellowes afford to our minds, an adequate explanation of the aggravation and epidemical extension of the usual endemic at Cadiz in 1800; while the gradual progress of the disease, and the imperceptible conversion of the ordinary and milder, into the more rare and and exalted form, constituting yellow fever, as manifested by the difficulties and dissentions which the Spanish physicians experienced in their attempts to fix the date, when the usual autumnal fever could be said to have ceased, and the epidemic yellow fever to have begun, confirm us in our opinion, that the question of Bulam, or continued Yellow Fever, is truly one of degree, and not of specific difference.

The author's former remarks on the defective signification of the term "marsh miasmata," to express the miasm of decomposition, are more especially applicable to the medical topography of Gibraltar, not unfrequently styled "par excellence" the Rock. The idea of the developement of paludal effluvia from a surface ostensibly so dissimilar to a marsh, has not merely been denied; it has been assailed by ridicule. The rarity of agues in Gibraltar has also been adduced in proof of the non-generation of those exhalations at that place. This, however, as the author shews, betrays a very limited acquaintance with the modifications which are impressed on endemic fever by the influence of locality; and while remittents are acknowledged to be the usual form of the autumnal fever in Gibraltar, (as well as in Cadiz,) we need take very little pains to prove the existence and influence of febrific exhalations from the soil, however ingeniously the speculators on the locality of an elevated rock, and on the absence of agues, may argue to the contrary. The examination of the importation account of the epidemic into Gibraltar in 1804, is prefaced by this observation.

"At present, therefore, it will be sufficient for me to suggest, as *obvious* and *prominent causes* of the epidemic in question, the accumulation of decomposable matters within the town, *and the long prevalence of a dry and scorching east wind*, which produced a very high atmospheric temperature, without any salutary ventilation of the place, as it was completely obstructed in its course by the high mountain behind the town, *in* and *over* which the air was for many weeks nearly stagnant. A similar dry and scorching east wind, blowing with too little force to change and purify the atmosphere, has invariably preceded and accompanied every recurrence of the yellow fever at Cadiz, and other cities of Spain. And its effects, in the year 1804, were very extensive and remarkable." p. 342—343.

We learn from the result of the enquiry into the alleged importation of that year, that Santos, the person who is accused of having imported the contagion into Gibraltar, from Cadiz, according to one account on the 28th of August, but according to another, on the 25th, left Cadiz several days *before* the time which Dr. Aregula, the chief official superintendant of all things belonging to the Andalusian epidemic, has declared to be the day on which the existence of the yellow fever was first discovered at Cadiz. He could not therefore have imported a disease from Cadiz which had no existence there. The importation by Santos, has been attempted to be corroborated by the evidence of a Mr. Pratt, who was also in Cadiz, and from whom Santos is alleged to have derived his contagion, while they resided in the same tavern. But the author says, that a very cursory view of his examination is sufficient to make any one " sensible of the obvious and irreconcileable contradictions which it contains, and of the absolute impossibility of its being true." The affidavit of this person states, that he was taken ill while living in a tavern in Cadiz, about the 18th or 20th of August; that eight days afterwards, he had symptoms of black or bloody vomiting; that then, fearful of being sent to an hospital, he removed to another part of the town, and ultimately recovered; and that *after* his recovery he applied for a passage to Gibraltar in the same vessel in which Santos returned to that place, but was refused on account of his very yellow look. The primâ facie improbability of a person who laboured under black vomit, being able to shift his quarters from the apprehension of any contingency, need not be insisted on; but the conclusion of the story is fatal to its credibility, and destroys all relation between the deponent's and Santos's illness; for the vessel in which Santos returned to Gibraltar, and in which Mr. Pratt says, he was refused a passage *after* his recovery, left Cadiz, *at the latest*, on the 24th of August, (as Santos and Sir James Fellowes assert, and public records prove,) *several days before* the occurrence of the alleged *black vomit* in the course of Mr. Pratt's illness.

From such a tissue of contradictions, we know not what points can be selected as entitled to belief. The statements intended to establish the fact of importation, reciprocally destroy their respective foundations. We, therefore, recur with unshaken confidence to the domestic origin of the epidemics; and proceed to shew, that the bases of the subsequent attempts to fix the mode of importation are equally deficient in solidity.

A coincidence of local and atmospherical causes, similar to those which produced the epidemic of 1804, again aggravated the usual remittent of Gibraltar (which had regularly prevailed there in every intermediate year,) towards the close of the autumn of 1810, to the degree of concentrated yellow fever. The epidemic of that year has also been alleged to have been imported by some transports from Carthagena, crowded with French deserters. The substantiability of this allegation may be in some degree appreciated by stating, that it rests wholly on the gratuitous assumption of a breach of quarantine. Some cases of fever had appeared among the soldiers in the transports, previous to their arrival at Gibraltar, of which one man had died. Sickness shortly ceased after their removal into hulks provided for their reception, and it does not appear that the fever was there communicated to any person; but the contagious nature of the disease was inferred from the subsequent attacks of the seamen, who remained in the transports, and of Mr. Arthur, who was sent on board them from the garrison to treat the sick. The cause of fever in those vessels, the author justly ascribes to the noxious emanations from their holds, which, in a former chapter, he has shewn to be capable of producing the worst yellow fevers. The attacks of Mr. Arthur and the seamen, are not proofs that the disease was contagious; the cause being local, every person exposed to its influence, might be expected to suffer, without the assumption of contagious agency. Dr. Bancroft refers to Dr. Burnett's previous statement in support of his rejection of the opinion of an imported contagion by these transports; but, it is necessary to repeat, that these vessels having been placed in strict quarantine immediately on their arrival at Gibraltar, the contagionists, in order to explain the origin of the epidemic by importation, are driven to the extremity of assuming a breach of quarantine. We would ask, if assumptions so perfectly gratuitous, be expected to be received as bonâ fide proofs of an affirmation, what fable, however preposterous, could be rejected on the score of want of evidence?

In the next epidemic in 1813, Sir Joseph Gilpin was at the head of the medical department in Gibraltar. In a letter to Dr. Chisholm, published in the Edinburgh Medical and Surgical Journal, in speaking of the contagious nature of yellow fever, and of its importation in 1793 from Africa into Grenada, he states, "of the infected state of the Hankey, I never did, nor ever shall, entertain the least doubt." This is certainly sufficiently declaratory of the tendency of his antecedent opinions. He says, that the first cases of the epidemic of 1813, occurred in two strangers, who imported it into Gibraltar on the 11th of August, in a vessel called the Fortune, from Cadiz, where he states (very erroneously, as will be shewn,)

the epidemic in question prevailed at the period of their departure. Now, Lieutenant General Campbell, the Lieutenant Governor of Gibraltar, writes to Sir James Duff, the British Consul at Cadiz, on the 13th of September, 1813, stating, that some cases of fever had lately occurred in the garrison, "but that there was not one of a contagious nature, as they were peculiar to the season only." Here we have the highest authority that no contagious disease prevailed in Gibraltar for more than a month after the arrival of the strangers from Cadiz; and the non-existence of the epidemic at Cadiz, not merely at the time of their departure from thence, but for a considerable time afterwards, is proved by the testimony of Sir James Fellowes, who, in speaking of Cadiz, states, at page 256, "in fact, until the end of August, the people collectively were, according to all the reports at the time, in a healthy state; and at page 261, he remarks, that it was only on the 14th of September that he observed any case in the British hospitals that excited his suspicions. These statements prove (as in the instance of 1804,) that no disease prevailed at Cadiz, at the time of the departure of the Fortune from that Port; she could not therefore, have imported a nonentity. Further, it has been seen, that more than a month elapsed after the arrival of the Fortune at Gibraltar, before the epidemic was observed in that garrison; on which point Dr. Bancroft observes,

"As Dr. Pym confidently asserts that the contagion of the Bulam produces disease *in four days*, at least in Gibraltar, its existence must have been made manifest by the occurrence of very many attacks within that interval; while, if it had been known to have produced *even one*, Sir Joseph Gilpin must have been highly culpable, had he not informed the Lieutenant Govenor thereof. p.375 376.

It is not a little curious, that "the garrison of Gibraltar was in strict quarantine *for several months before* the malady made its appearance, and a Board of Health was sitting *almost daily* on account of the plague which had broken out at Malta."

This circumstance, added to the *assumed* breach of quarantine in 1810, inevitably involves the dilemma, of either acknowledging the futility of quarantine regulations for the prevention of the Bulam; or otherwise, that the disease was not in either case imported. The advocates for quarantines are at liberty to choose their difficulty, — the impossibility of supporting both positions is palpable.

The origin of the epidemic of 1814, the last which has occurred in Gibraltar, has not been attempted to be referred to importation, except by one individual, who advances no facts in support of his opinion. By the replies to the questions produced by Deputy Inspector Fraser to the medical officers of the garrison, seventeen in number, we learn, that twelve declared it to be their belief, that the disease originated in domestic or local causes, unconnected with importation. Three were neutral; one declined offering an opinion; and one only derived it from importation. The original documents adduced in proof of the domestic origin of the epidemic of that year, are too numerous for us even to glance at. We, therefore, take our leave of the subject of yellow fever at Gibraltar, by repeating our perfect concurrence with the author, after a deliberate consideration of the question, that the fever which has prevailed there epidemically several times within the present century, originated from local or domestic causes, and was destitute of any contagious property.

The seventh and last chapter contains an inquiry into the causes of the epidemics of Cadiz, and other places in Spain in 1810, and in some subsequent years; but, as the disease was avowedly the same with that of former periods, it will not be incumbent on us to notice all the particular subjects, which, in order to leave nothing relating to these epidemics without investigation, Dr. Bancroft has deemed it his duty to examine. With respect to the fever of Carthagena in 1810, which caused the deaths of 3000 persons in six or eight weeks, he observes,

"We are told by Dr. Burnett, (p. 274) that Dr. Risneno, physician to the Spanish Royal Hospital there, 'positively asserts, that the fever was brought from Cadiz and Gibraltar, in 1810;' while Dr. Pym as positively asserted it to have been carried from Carthagena to Gibraltar. This last assertion has already been proved tob e erroneous (see page 359, &c.); and the former must be so, because the ardent yellow fever, or Bulam, did not appear at Gibraltar (except in the transports), until near the middle of October, a month after the disease had been prevalent in Carthagena; and this observation is also applicable to Cadiz, which continued healthy till the middle of September, 'before which time many deaths had occurred at Carthagena;' and these contradictory assertions serve only to manifest the readiness with which the contagionists, who believe that an epidemic yellow fever must always proceed from imported contagion, hazard tales to account for it." p. 415.

The history of an epidemic yellow fever, which prevailed in the 54th regiment at Stony Hill in Jamaica, has been brought forward by Dr. Pym, as a proof of the contagious origin of that disease. The opinion rests on the circumstance, that a detachment of the 54th regiment, which was sent from Stony Hill to Fort Augusta, and there quartered with a Negro regiment and some European troops, became sickly; and that after their return to Stony Hill, fever passed through the whole regiment. It is not said from *whence* the contagion was derived; certainly not from the Negroes at Fort Augusta, who know nothing of yellow fever; nor yet from the European troops in those quarters; for it is stated, that the other regiments in the same quarters with the detachment of the 54th, were not affected by the fever. "If therefore no contagion existed in Fort Augusta, none could have been carried to Stony Hill."

This statement had already been controverted by Mr. Doughty in his valuable publication on Yellow Fever.

"That the 54th Regiment was attacked with the aggravated form of yellow fever, as described in these letters (published by Dr. Pym), I readily admit; and, that the other corps in the same quarters did not suffer, as stated by Mr. Rocket, I also most firmly believe. Now, as Mr. Redmond and Mr. Pym agree that the fever which prevailed in the 54th regiment was highly contagious, and Mr. Rocket asserts the other corps remained unaffected with it, I ask from what source did the 54th imbibe its contagion? The fever developed itself at the season when the endemic cause prevailed, and which might that year be more powerful, and exert its influence to a wider extent, than it had done the preceding years. The soldiers of the 54th were susceptible of its influence, whilst those of the other corps were not, in the same degree; because one of those latter regiments had been in the island, to my knowledge, not less than *three*, and the other *six* years, and a great part of the time in quarters, annually visited with yellow fever." *Observations on Yellow Fever.* p. 54.

There remains the history of another epidemic yellow fever, recorded by Dr. Pym as owing its origin to contagion, which we are somewhat surprized to find that Dr. Bancroft has omitted to notice; more especially as his local knowledge of the scene of the transaction (with which we also have some acquaintance), would, we apprehend, have rendered the task of its refutation void of difficulty. We allude to the fever of the 70th regiment in Fort Edward, Martinique, in 1794; and refer to Dr. Fergusson's excellent topographical remarks on Fort Royal; (Med. Chir. Trans. Vol. viii, p. 119 to 122.) and also to Mr. Mortimer's introductory letter to his valuable report on Yellow Fever, published in this Journal, in proof, that the sickness of that regiment, attributed by Dr. Pym to contagion, (Obs. on the Bulam Fever, p. 10—14) depended wholly upon local and indigenous causes.

We conclude this subject with the author's exhortation respecting the preconceived opinions of contagion, which Strangers usually carry with them into tropical climates; but which, in by far the majority of instances, ultimately yeild to a more intimate acquaintance with the habitudes of the disease in question.

"I earnestly request my readers attentively to reflect upon the facts stated in this chapter; and especially upon the readiness with which numerous medical men, respectable by their characters, their conduct, and their professional ranks, have come forward to make confessions which are generally felt as in some degree humiliating, by acknowledging, that they had, when they first arrived in the regions of yellow fever, entertained opinions, deeply fixed in their minds by the ordinary course of medical education, which however, after more extensive observation and better means of information, they had found reason to abandon as erroneous, and been forced to adopt conclusions directly the reverse, in regard to the alleged contagious nature of the yellow fever. This is stated to have been done by Dr. M'Lean, Dr. Fergusson, and all their colleagues on the hospital staff at St. Domingo; it was done also by myself, and almost all on the hospital staff in the Windward Islands (see the letter of Mr. Young, Inspector General, on this subject, at page 335 of my Essay); it was done by Dr. Dickson, and, as he declares, generally by others in the circle of his acquaintance; and, beside many others, it will soon appear to have been done by Dr. Erly at Sierra Leone, on the very coast where Dr. Pym and Dr. Chisholm pretend to derive their Bulam fever. In all these cases, the change of opinion has been made spontaneously and disinterestedly, by the silent and gradual, but certain operation of truth; and without any desire to gain credit by a supposed preservation of many lives from a danger which had no existence, and without any of those views to promotion and reward, which may have produced some of the exertions and erroneous statements lately made, in regard to the fever under consideration." p. 189—191.

On the subject of typhus within the tropics, we think Dr. Bancroft has somewhat, and with advantage, modified his former opinions; for his admission, p. 174, seems to sanction a greater latitude of inference, than could be deduced from his former volume, respecting its being carried as far as Barbadoes. We also are of opinion, that typhus may, and does exist occasionally within the tropics; and we have seen what we considered to be unequivocal cases of that disease on the Atlantic Equator; but we coincide with the author, that the climate is extremely unfavourable to the existence and perpetuation of typhus contagion, and that it ultimately exhausts itself.

Upon the occasional occurrence of a hybrid disease, which Dr. Bancroft simply alludes to as having noticed in his Essay "without either approbation or disapprobation," we do not profess to offer any decided opinion. It is known, that Sir John Pringle, Sir Gilbert Blane, Dr. Lempriere, and others, have spoken of a mixed or hybrid fever; and we have understood, that Dr. Dickson is of opinion, that he has seen some instances which favour the existence of such character of Disease; where the appearance and duration of the symptoms were so intermediate between typhus and yellow fever, that it was difficult to say, to which order of fever they most belonged. But we believe at the same time, that he considers such occurrences as extremely rare; that he has not detected any satisfactory evidence of their possessing an infectious quality; and, that under the influence of climate, they soon disappear, and are succeeded by the legitimate endemic of the West Indies. Such questions can only, we conceive, be ultimately decided by those who may enjoy similarly extensive opportunities of witnessing the disease under all varieties of circumstances and character.

We conclude by expressing our sense of the ingenuity, acuteness, and research, which the author has exerted with equal felicity and effect in the present elaborate production; and we are satisfied, that the voluminous mass of irrefragable evidence which he has been enabled to adduce, will impress conviction on every unprejudiced mind, of the perfect triumph he has achieved by the complete refutation of the opposite opinion, of the existence of the Bulam as a distinct contagious fever, attacking but once. In the preceding analysis, we have aimed at the inclusion of the most prominent parts of the discussion; for its length we plead the importance of the inquiry, and the desire to diffuse a portion, at least, of the information with which the pages of this Sequel are enriched, as well as to contribute our mite to the advancement of what we consider to be the cause of truth, and to the correction of a popular error; for as the author justly observes in his conclusion, the supposition of the existence of contagion "accords with the prejudices and apprehensions of the greater part of mankind, who are prone to believe that all diseases are contagious when they become generally prevalent." To those whose lot and duty it has been to alleviate the sufferings inflicted by yellow fever, and who, therefore, with us, naturally feel a peculiar interest in the discussion, we need not say more to induce them to avail themselves of the information and experience accumulated in this volume. To the profession at large we recommend an attentive consideration of the ætiological principles inculcated therein, as connected generally with the science of medicine; while we feel assured, that the acknowledged talents and experience of Dr. Bancroft, and the infinite preponderance of the facts he has adduced in proof of yellow fever being destitute of contagion (as well as indisputably establishing the liability to repeated attacks), over those of a contrary tendency, will be duly appreciated by the higher official powers, in the regulation of their future measures, relative to a subject of the highest importance to the best interests of our commercial empire.

